# Lactylation as a metabolic–epigenetic switch: Mechanisms and roles in cancer, sepsis, trauma, inflammation, and tissue repair

**DOI:** 10.1016/j.bbrep.2026.102507

**Published:** 2026-02-16

**Authors:** David Bar-Or, Kaysie Banton, David Acuna, Jason Williams, Carlos H. Palacio, Christopher Zaw-mon, Raymond Garrett, Tyler Crawley, Daniel Paredes

**Affiliations:** a Trauma Services, Swedish Medical Center, Englewood, CO, USA; b Trauma Services, McAllen Hospital, McAllen, Texas, USA; c Trauma Services, Wesley Medical Center, Wichita, KS, USA; d Trauma Services, Lutheran Hospital, Wheatridge, Colorado, USA; e Trauma Research, Swedish Medical Center, Englewood, CO, USA; f Department of Chemistry and Biochemistry, University of Denver, Denver, CO, USA

**Keywords:** Lactate, Lactylation, Inflammation, Trauma, Sepsis, Metabolic, Epigenetics

## Abstract

Lactylation, a recently discovered post-translational modification, links cellular metabolism to epigenetic regulation and immune function. Once considered a mere glycolytic byproduct, lactate is now recognized as a signaling metabolite that can shape gene expression and protein activity via histone and non-histone lactylation. This review synthesizes evidence on enzymatic and non-enzymatic mechanisms of lactylation, including lactoyl-CoA-dependent pathways, glyoxalase-mediated routes, and emerging functions of aminoacyl-tRNA synthetases. We highlight lactylation “writers” and “erasers,” stereochemical considerations, and integration with other acyl modifications while explicitly distinguishing well-supported mechanisms from hypothesis-generating findings. Functionally, lactylation influences inflammatory signaling, metabolic reprogramming, and immune cell polarization, providing a conceptual link across sepsis, trauma, autoimmune disease, cancer, and tissue repair. Examples include HMGB1 modification cascades in sepsis, macrophage state transitions in inflammation, fibroblast/angiogenic programs in wound environments, and DNA repair regulation in cancer drug resistance. The context-dependence of lactylation adaptive versus pathological, tumor-promoting versus potentially tumor-restrictive underscores the need for temporal, cell-type, and compartment-specific interpretation. We conclude with methodological constraints, translational feasibility, and key priorities for moving lactylation from a mechanistic bridge to a clinical biomarker and therapeutic axis.

## Highlights

• Lactate acts as a signaling metabolite influencing gene expression via lactylation.
• Lactate modulates immune responses via lactylation in macrophages and other cells.
• Lactate-driven lactylation influences tumor microenvironment and cancer progression.
• Targeting lactylation pathways offers new therapeutic strategies in disease treatment.

## 1 Introduction

This review re-evaluates the traditional understanding of lactate and introduces protein lactylation as a post-translational modification that couples metabolic state to chromatin regulation and cellular signaling.

Sepsis is a critical condition characterized by life-threatening organ dysfunction that emerges from a dysregulated host response to infection. This concept was refined in the 2016 Sepsis-3 definitions, which shifted the diagnostic focus from SIRS to organ dysfunction metrics such as SOFA and qSOFA [1]. The insight that sepsis reflects dysregulated host response rather than inflammation alone highlights the need to understand metabolic-immune coupling mechanisms that shape disease trajectories.

Trauma elicits a systemic inflammatory response and immune dysregulation that overlaps with sepsis biology across the acute and recovery phases. Evidence suggests both conditions share metabolic reprogramming and immune maladaptation that contribute to mortality and long-term morbidity [[2], [3], [4]]. This overlap motivates investigation of molecular “bridges” between metabolism and immune/epigenetic regulation, including lactate-dependent signaling and covalent modifications such as lactylation.

Investigations into cytokines, immune activation, and metabolic signaling may illuminate shared pathophysiology across critical illness contexts and guide intervention strategies aimed at reducing organ failure and long-term complications.

### 1.1 Lactate: Re-evaluating its role beyond a metabolic byproduct

The understanding of lactate has evolved substantially. Historically, lactate was viewed as a byproduct of anaerobic glycolysis, generated from pyruvate via lactate dehydrogenase (LDH). However, lactate is now recognized as a signaling metabolite with multifaceted roles across metabolism, immune regulation, and gene expression [5]. In critical illness, lactate can function both as an indicator of metabolic stress and as a mediator capable of influencing disease trajectories through downstream signaling and covalent protein modifications [6].

Lactate is not confined to hypoxic conditions; it can be produced through the lactate shuttle and signaling-associated glycolysis, even in aerobic states [7,8]. It is also a hallmark product of the Warburg effect in cancer, where aerobic glycolysis yields high lactate despite oxygen availability, shaping the tumor microenvironment and potentially facilitating lactylation-linked transcriptional reprogramming [8].

Importantly, extracellular lactate can also signal through receptor-mediated pathways (e.g., GPR81) and through transport-driven changes in intracellular metabolite pools. These mechanisms are conceptually distinct from lactylation and are explicitly separated later to avoid conflation (Section 5.2).

### 1.2 The discovery and significance of protein lactylation

The concept that small metabolites can regulate epigenetic processes is well established, exemplified by histone acylation [9]. In 2019, Zhang et al. identified histone lysine lactylation (Kla) as a novel PTM [10] detected by HPLC-MS/MS as a 72.021 Da mass shift on lysine residues. Xie et al. also reported lactate-linked histone lactylation and transcriptional modulation [11]. These findings provide a molecular link between cellular lactate levels and the epigenetic landscape [12,13], with relevance to diseases characterized by metabolic dysregulation such as cancer [14,15]. It emphasizes lactylation as a prime target for novel epigenetic therapies aimed at restoring metabolic and transcriptional homeostasis.

Initial research proposed two distinct mechanisms for lactylation: an enzymatic process dependent on lactoyl coenzyme A (Lactoyl-CoA) as a substrate, speculated by Zhao et al., and a passive, non-enzymatic acyl transfer utilizing lactoyl glutathione (LGSH) as a substrate [16].

The distinction between these pathways signifies not just two routes leading to the same outcome but also indicates different regulatory inputs and potentially distinct biological contexts. For example, the non-enzymatic pathway is influenced by levels of methylglyoxal and activity of glyoxalase enzymes, directly linking it to metabolic stress. This mechanistic duality suggests that lactylation is a highly adaptable modification, capable of responding to both enzymatic machinery and direct metabolic flux. Understanding which mechanism predominates in specific disease states could guide the development of more precise and effective therapeutic interventions.

Subsequent studies have demonstrated that lactylation leads to profound changes in gene expression via epigenetic regulation. Lactylation modifications can also provide feedback regulation on various enzymes, particularly within metabolic pathways. The dynamic nature of lactylation is governed by specific "writer" enzymes (lactyltransferases) that add lactyl groups and "eraser" enzymes (delactylases) that remove them. Early identified writers included p300 [17], while erasers included HDAC1 and HDAC2 [18]. The versatility of lactylation, particularly in its role as a mediator of metabolic reprogramming and immune responses, notably underscores its potential as both a biomarker and therapeutic target in various pathological conditions.

### 1.3 A unifying framework: When is lactylation adaptive versus pathological?

The literature spans diverse conditions and can read as fragmented unless anchored to a shared conceptual model. We therefore propose a context-dependent framework in which lactylation can be adaptive (supporting timely resolution, tissue restoration, or metabolic transitions) or pathological (persisting in a way that locks cells into maladaptive immune suppression, chronic inflammatory signaling, fibrosis-like remodeling, or therapy resistance).

**Fig. 1:**
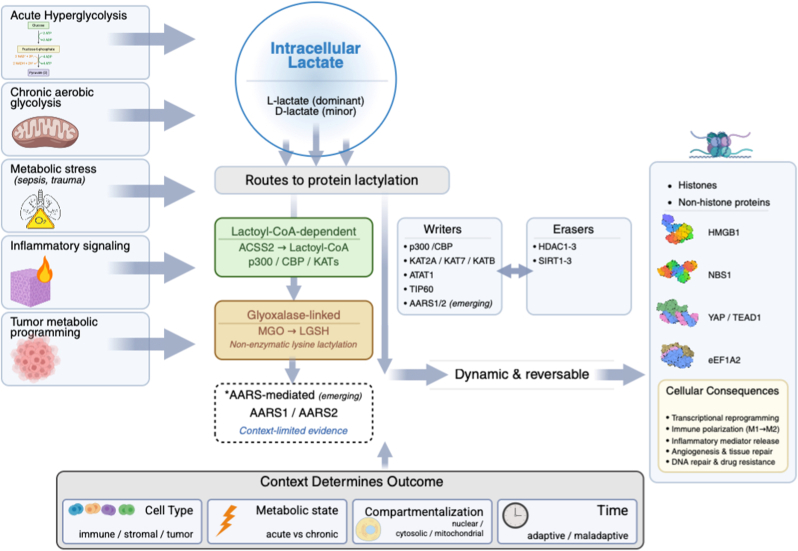
**Lactylation as a context-dependent metabolic–epigenetic switch**. Elevated glycolytic flux and lactate accumulation (sepsis, trauma, inflammation, wound repair, cancer) influence cells via receptor-mediated signaling, transport-dependent metabolic effects, and covalent protein lactylation. Lactylation can arise through lactoyl-CoA–dependent enzymatic pathways (e.g., ACSS2-coupled transferases, p300/CBP, KATs), glyoxalase/lactoyl-glutathione–linked non-enzymatic routes, and emerging lactoyl-CoA–independent AARS mechanisms, counterbalanced by delactylases (HDAC1–3, select sirtuins). These modifications reshape histone and non-histone programs controlling transcription, immune polarization, inflammatory mediator release, angiogenesis, DNA repair, and survival. Net effects depend on cell type, metabolic state, compartmentalization, and timing—often adaptive in acute stress but maladaptive when sustained.

Four variables shape this balance: (1) cell type/state (immune, stromal, endothelial, tumor; polarization and differentiation programs), (2) metabolic context (acute stress versus chronic glycolytic bias), (3) lactate burden and compartmentalization (intracellular pools versus extracellular microenvironment), and (4) time (early adaptation versus persistent “memory” states) Fig. 1.

Throughout Sections 1.3, 2, 2.1 we map disease-specific findings onto these variables and explicitly label conclusions as mechanistic/causal versus associative/hypothesis-generating where appropriate.

## 2 Biochemical mechanisms of lactylation

### 2.1 Lactoyl-CoA dependent enzymatic lactylation

Lactoyl-CoA, a crucial substrate for enzymatic lactylation, is synthesized through the activation of lactic acid by coenzyme A (CoA). This reaction is typically catalyzed by specific enzymes, such as lactate-CoA ligase. The chemical reaction can be summarized as: Lactic Acid + CoA + ATP → Lactoyl-CoA + AMP + PPi, with ATP providing the necessary energy for this activation [19,20].

Quantitative analyses indicate lactoyl-CoA is present in mammalian tissues at concentrations substantially lower than more prevalent acyl-CoAs [20], suggesting compartmentalization and tight regulation; small fluctuations could therefore have disproportionate effects on lactylation dynamics in permissive settings.

The presence of L- and d-lactate is noteworthy: l-lactate predominates in serum while d-lactate is influenced by diet and microbiome contributions [21]. While stereospecific lactylation is plausible, direct evidence that L-versus D-lactylation produces distinct functional outcomes in vivo remains limited; accordingly, stereospecificity is discussed here as a promising but still maturing concept rather than an established determinant.

#### 2.1.1 Identification of mammalian Lactoyl-CoA synthetases

Recent studies have identified specific enzymes responsible for synthesizing lactoyl-CoA in mammalian cells. Acetyl-CoA synthetase 2 (ACSS2) has been identified as a bona fide lactoyl-CoA synthetase. ACSS2 converts lactate to lactoyl-CoA, which subsequently binds to lysine acetyltransferase 2A (KAT2A). This ACSS2-KAT2A coupling functions as a lactyltransferase system for histone lactylation, thereby influencing important gene expression pathways such as Wnt/β-catenin and NF-κB, which can promote brain tumor growth and immune evasion [22,23]. The identification of at least two distinct mammalian lactoyl-CoA synthetases, specifically ACSS2, suggests that lactoyl-CoA production and enzymatic lactylation are regulated in a context-dependent and spatially controlled manner [18,23].

Different enzymes may respond to various metabolic cues or be activated in specific cellular compartments to serve distinct functional needs. This complexity implies that therapeutic strategies targeting lactoyl-CoA synthesis for modulating disease might require consideration of specific cellular contexts, for example, targeting ACSS2 in glioma, as opposed to other cancers, enhancing therapeutic efficacy and minimizing off-target effects. Lactyl glutathione (LGSH) has also been proposed as a metabolic precursor for D-lactoyl-CoA, suggesting a potential connection between the enzymatic and non-enzymatic pathways for synthesizing specific lactoyl-CoA enantiomers [24].

### 2.2 Non-enzymatic lactylation via the glyoxalase pathway

Lactylation can also occur through a non-enzymatic mechanism involving the glyoxalase pathway and the reactive glycolytic byproduct methylglyoxal (MGO) [25]. The intracellular concentrations of MGO are regulated by the glyoxalase cycle, which comprises two key enzymes: glyoxalase 1 (GLO1) and glyoxalase 2 (GLO2) [26]. GLO1 facilitates the isomerization of the glutathione (GSH)-MGO hemithioacetal, leading to the production of S-d-lactoylglutathione (LGSH). Subsequently, LGSH is hydrolyzed by GLO2, recycling GSH and generating d-lactate [26]. This sequence positions GLO2 as a critical regulator within this pathway, where the knockout of GLO2 markedly elevates LGSH levels, resulting in a significant increase in lactylation [27].

The non-enzymatic acyl transfer of the lactate moiety from LGSH directly to protein lysine residues facilitates the formation of "LactoylLys" modifications. This relationship establishes a direct link between a glycolytic byproduct (MGO) and protein modification through LGSH, with GLO2 acting as a key regulator [28]. The observed augmentation in lactylation following GLO2 knockout illustrates a feedback mechanism in which metabolic dysregulation, such as heightened levels of MGO due to hyperglycemic conditions can catalyze non-enzymatic lactylation. This indicates a rapid, enzyme-independent response to metabolic shifts [25].

Moreover, this pathway offers a unique regulatory node for lactylation that operates independently of specific lactyltransferase enzymes. Consequently, conditions that induce increased MGO or impede GLO2 activity could promote widespread non-enzymatic lactylation, potentially affecting various metabolic enzymes and contributing to a self-regulatory or dysregulatory loop, particularly relevant in metabolic disorders and cancer [29]. These non-enzymatic modifications are notably enriched on glycolytic enzymes, suggesting they may partake in feedback mechanisms that regulate glycolytic flux in states of hyperglycemia [30].

### 2.3 Lactoyl-CoA independent enzymatic lactylation: the emerging roles of Aminoacyl-tRNA synthetases

Recent work proposes a lactoyl-CoA independent pathway in which aminoacyl-tRNA synthetases (AARS), specifically AARS1/2, can catalyze lactylation via a lactate-AMP intermediate [31]. In a gastric cancer context, AARS1 has been reported to lactylate TEAD1, TP53, and YAP1, influencing Hippo signaling [31].

At present, supporting evidence is concentrated in specific cancer models, and generalizability across immune-cell biology, sepsis/trauma physiology, or tissue repair remains uncertain. We therefore present AARS-mediated lactylation as a hypothesis-generating mechanism outside the contexts in which it has been directly demonstrated.

## 3 Regulatory enzymes of lactylation: writers and erasers

### 3.1 Lactyltransferases ("Writers")

Lactyltransferases catalyze addition of lactyl groups to lysine residues. Many candidates overlap with acetyltransferase families (p300/CBP, KAT2A, HBO1/KAT7, KAT8, ATAT1, TIP60), suggesting competition or coordination with acetylation on shared lysines and shared cofactor constraints [23]. This overlap suggests that the enzymatic machinery for lactylation may share structural and functional similarities with the acetylation machinery, potentially leading to competition for shared lysine residues on target proteins or coordinated regulation between these two post-translational modifications (PTMs). This dynamic interplay represents a crucial regulatory layer in cellular responses to metabolic changes and disease progression, offering therapeutic avenues through the targeting of enzymes with dual specificity.

**p300/CREB-binding protein (CBP)** has been identified as a key enzyme responsible for histone lactylation. p300 exhibits a high affinity for lactoyl-CoA, and its overexpression increases histone lactylation in cells such as HEK293T. Conversely, the knockdown of p300 results in decreased lactylation of HMGB1. Notably, lactate can stimulate the nuclear translocation of p300/CBP, enhancing its interaction with HMGB1, which promotes HMGB1 lactylation and release in macrophages during inflammation [23].

**Acetyl-CoA Synthetase 2 (ACSS2)** functions as a lactoyl-CoA synthetase, and its product, lactoyl-CoA, can act as a substrate for lysine acetyltransferase 2A (KAT2A). This coupling acts as a lactyltransferase system for histone H3 lactylation, regulating gene expression pathways such as Wnt/β-catenin, NF-κB, and PD-L1, thereby promoting tumor growth and immune evasion [32].

**HBO1 (KAT7)** catalyzes lysine lactylation, specifically of histone H3K9, influencing gene transcription and is implicated in tumorigenesis [33].

**KAT8**, identified as a lactyltransferase, catalyzes the lactylation of eEF1A2 at lysine 408 (eEF1A2K408). This modification enhances translation elongation and increases protein synthesis, thus promoting tumor progression, particularly in colorectal cancer [34,35].

**Alpha-tubulin acetyltransferase 1 (ATAT1)** has previously been considered solely responsible for alpha-tubulin acetylation; however, it has now been shown to also mediate lactylation of N-acetyltransferase 10 (NAT10), which promotes N4-acetylcytidine modification on tRNAs, facilitating oncogenic DNA virus KSHV reactivation [29].

**Aminoacyl-tRNA Synthetases (AARS1/2)**: As discussed in Section 2.3, AARS1 and AARS2 have been suggested to catalyze the lactylation of target proteins such as TEAD1, YAP1, and p53 independently of lactoyl-CoA [30]. However, further evidence is needed to fully substantiate these interactions.

**TIP60** has a role as a mediator of lactylation at K388 of NBS1, enhancing homologous recombination-mediated DNA repair and increasing chemotherapy resistance, is supported by the study conducted by Chen et al. [36]. This research indicates that lactate-driven lactylation of NBS1 at lysine 388 (K388) is essential for the efficient formation of the MRE11–RAD50–NBS1 (MRN) complex. This lactylation promotes the recruitment and accumulation of homologous recombination (HR) repair proteins at the sites of DNA double-strand breaks (DSBs), thus contributing to enhanced DNA repair mechanisms and resistance to chemotherapy in cancer cells. Furthermore, the authors identify TIP60 as the specific enzyme responsible for the lactylation of NBS1, establishing it as a critical "writer" for this modification.

Collectively, these studies support the view that lactylation extends beyond chromatin to modify signaling and structural proteins; however, mechanism dominance and in vivo relevance vary by tissue and disease (summarized in Table 1).

**Table 1:** **Context-dependent lactylation across disease states.** For each clinical context, the table summarizes the dominant metabolic milieu, the most likely lactylation route(s), representative modified targets, functional interpretation, strength of evidence, and translational relevance.

Disease context	Dominant metabolic context	Likely lactylation route(s)	Key modified targets (examples)	Functional role of lactylation	Evidence tier∗	Translational interpretation
Sepsis	Acute hyperglycolysis, high lactate burden	Enzymatic (p300/CBP–dependent); LGSH-linked non-enzymatic	HMGB1, histones	Amplifies inflammatory signaling, NET formation, organ injury	Causal (cellular/animal models)	Disease modulator; candidate biomarker of severity
Trauma/Critical illness	Acute metabolic stress, fluctuating lactate	Likely enzymatic; poorly resolved	Not yet well defined	Hypothesized immune trajectory modulation	Associative/emerging	Mechanistic hypothesis; biomarker potential
Inflammatory & autoimmune disease	Chronic glycolytic bias in immune cells	Enzymatic histone lactylation	Histones (macrophage genes)	Shapes macrophage polarization (M1 ↔ M2)	Mixed (causal + associative)	Context-dependent modulator
Wound healing	Local lactate accumulation, reparative metabolism	Permissive for enzymatic lactylation	Not well defined	Supports reparative transcriptional programs	Associative	Emerging biology; indirect evidence
Cancer	Persistent aerobic glycolysis (Warburg effect)	Enzymatic (ACSS2-KAT, AARS-mediated); some non-enzymatic	Histones, YAP/TEAD1, NBS1, eEF1A2	Promotes immune evasion, DNA repair, drug resistance (context-dependent)	Causal (tumor models)	Therapeutic target (context-specific); biomarker of resistance
Fibrosis-like remodeling	Chronic metabolic reprogramming	Enzymatic histone lactylation	Histones	Sustains maladaptive transcriptional memory	Associative	Disease progression marker

### 3.2 Delactylases ("Erasers")

Delactylases are enzymes responsible for removing lactyl groups from proteins, rendering lactylation a reversible and dynamically regulated post-translational modification (PTM). The shared enzymatic machinery for removing acetyl and lactyl groups, including histone deacetylases (HDACs) and sirtuins, implies a coordinated regulatory network between these PTMs [18]. This suggests that modulating these enzymes could have pleiotropic effects on cellular signaling and disease. The opposing actions of "writers" and "erasers" enable precise control over lactylation levels, allowing cells to adapt to metabolic and environmental changes. This dynamic control is essential for cellular homeostasis and presents opportunities for therapeutic intervention by tipping the balance of these modifications.

However, much of the evidence for delactylation derives from in vitro or cellular assays, and in vivo disease-model validation of specific delactylase dependencies remains limited in many contexts; therefore, delactylation is treated here as a promising regulatory axis that requires broader physiologic validation.

Histone Deacetylases (HDACs) are classified into four classes (Class I–IV) based on their structure and cofactors. Zn^2+^-dependent enzymes (Classes I, II, and IV) include HDAC1–11. Class I enzymes (HDAC1–3, 8) are primarily reported as delactylases and are predominantly localized in the nucleus. Class II enzymes (HDAC4–7, 9–10) and Class IV enzyme (HDAC11) typically shuttle lactoyl-CoA between the nucleus and cytoplasm. Studies have identified HDAC1–3 and sirtuins (SIRT1–3) as delactylases in vitro, providing insight into their roles in protein modification dynamics [18].

SIRT1, a NAD^+^-dependent enzyme, has been shown to remove lactylation from α-myosin heavy chain (MHC), thereby protecting myocardial structure and function in heart failure [37]. SIRT2 serves as an efficient "eraser" of multiple histone lactylation sites, targeting synthetic histone peptides, purified histones, nucleosomes, and histones in cancer cells [38]. However, the delactylation capacities of other SIRT family members remain poorly understood, necessitating further investigation.

It's crucial to note that some HDAC family members exhibit multifunctionality, acting both as delactylases and deacetylases. For instance, HDAC1 has demonstrated functionality in both processes, indicating that the regulation of lactylation may not be independent of acetylation dynamics [39]. This interplay suggests that the actions of delactylases could influence broader cellular pathways, particularly under conditions of metabolic stress or in disease states, such as cancer, where alterations to protein modifications can drive tumorigenesis [40]. Understanding the functional implications of delactylation by these enzymes may reveal new therapeutic targets in metabolically dysregulated conditions, enhancing strategies that leverage the intricate links between lactylation and cellular signaling cascades.

## 4 Lactylation outcomes: Physiological and Pathological roles

### 4.1 Broad cellular processes influenced by lactylation

Lactylation influences a wide range of cellular processes, including gene expression, metabolism, tumorigenesis, and immune response. This broad impact underscores its critical role in cellular regulation, making lactylation a central player in both health and disease. Both histone and non-histone proteins are susceptible to lactylation.

Lactate itself has been identified as a key determinant of histone lactylation levels. Studies on Trypanosoma brucei indicate that alterations in lactate concentration significantly affect histone and total protein lactylation [41]. Additionally, lactate promotes the lactylation of HMGB1 in macrophages, linking lactate to immune responses during sepsis through mechanisms that modulate gene transcription [42]. This discovery emphasizes lactylation's role in enhancing gene expression related to immunity and influencing chromatin structure.

Moreover, lactylation has been implicated in cancer progression. Research indicates that lactylation can modify histones, alter chromatin conformation and affect DNA accessibility, thereby playing a role in tumor microenvironment reprogramming [43]. The lactylation of non-histone proteins, such as the mitochondrial protein YTHDF2, has been shown to drive oncogenic processes in ocular melanoma by facilitating specific mRNA modifications [14].

Recent studies have highlighted the prognostic significance of lactylation-related gene signatures in hepatocellular carcinoma. These studies link lactylation to tumorigenesis and immune evasion mechanisms [44]. Additionally, lactylation has been suggested to enhance mesenchymal stem cell histone modifications, allowing for the modulation of bone density, which could impact conditions such as osteoporosis [45].

The influence of lactylation extends to various physiological and pathological processes, including inflammatory responses, metabolic reprogramming, and cell signaling. For example, the connection between lactylation and inflammatory macrophage activation highlights its role as a modifier of immune cell function, integrating metabolic changes with immune signaling pathways [46].

### 4.2 Health and disease contexts

#### 4.2.1 Sepsis

Sepsis-induced metabolic reprogramming favors aerobic glycolysis, leading to lactate accumulation. Lactate has emerged as a significant player in promoting lactylation and acetylation of High Mobility Group Box 1 (HMGB1) proteins via exosomal secretion in macrophages [47]. HMGB1, a critical damage-associated molecular pattern (DAMP), plays a pivotal role in the progression and late mortality of sepsis [[48], [49], [50], [51]]. The post-translational acetylation of lysine residues in HMGB1 has been identified as a primary mechanism underlying HMGB1 release during sepsis [48,52]. Lactylation may provide additional regulatory nuance by introducing lactate-sensitive modification states that coexist with acetylation and may influence timing, localization, or magnitude of HMGB1 export, rather than replacing acetylation as the central mechanism.

Lactate-induced HMGB1 lactylation has been linked to NET formation and exacerbation of acute kidney injury in sepsis models [53]. supporting a plausible pathogenic role in late inflammatory cascades. Lactylation has also been proposed as a biomarker axis in septic shock contexts [51], but practical feasibility constraints are addressed in Section 5.3.

The dynamic interplay between lactate and HMGB1-related signaling pathways demonstrates how metabolic variations can influence inflammatory responses and worsen organ injury conditions associated with sepsis. Lactylation reflects a direct mechanism by which alterations in lactate levels can impact the physiological processes that govern cellular function and pathology in the context of sepsis [54].

Overall, the sepsis literature supports lactylation as a mechanistically plausible modulator/driver in select pathways, with timing likely determining whether lactylation is adaptive early versus maladaptive if sustained (framework in Section 1.3).

#### 4.2.2 Inflammatory immune diseases

Lactate can amplify or inhibit inflammation depending on concentration and cellular context. In macrophages, lactate can suppress inflammatory cytokines and inhibit NF-κB signaling through pathways involving GPR81 signaling [55]. These receptor-mediated effects are mechanistically distinct from covalent lactylation and should not be conflated.

Macrophage polarization depends on microenvironmental stimuli and metabolic state. Lactate can support M2-like polarization in dose- and context-dependent ways [56], and histone lactylation has been implicated in inflammatory to resolution macrophage transitions [56,57].

Chronic inflammation is characterized by increased infiltration of T lymphocytes and macrophages, with lactate enhancing this process. It has been observed that lactate prolongs chronic inflammation by inhibiting the migration of T cells, thereby retaining them at sites of inflammation and increasing the production of inflammatory cytokines while reducing the rate of cell lysis [58,59].

The overexpression of lactate dehydrogenase (LDH), responsible for lactate formation, has been noted in CD8^+^ T-cells in rheumatoid arthritis contexts. Conversely, inhibiting LDH in rheumatoid arthritis can alleviate inflammatory effects exerted by these T-cells [60] chronic inflammation while inhibiting acute inflammatory responses, emphasizing the complex duality of its role depending on concentration and cellular context [61,62].

Together, these findings support lactate and lactylation as candidate regulators of immune states, but the degree to which lactylation is an upstream driver versus a downstream consequence remains context-dependent and requires careful disease-model validation.

#### 4.2.3 Wound healing

Wound healing proceeds through hemostasis, inflammation, proliferation, and remodeling. Lactate accumulates in wounds and serves as an energy substrate while influencing pH and redox conditions that support cell proliferation and differentiation [63]. Lactate promotes angiogenesis via VEGF-associated pathways and endothelial migration [64,65] and stimulates fibroblast proliferation and collagen-related programs [66,67].

Much of the wound literature describes lactate biology broadly (energy provision, pH modulation, angiogenesis, collagen synthesis). Direct evidence that specific lactylation marks (histone or non-histone) causally drive wound repair remains comparatively limited; therefore, lactylation in wound repair should currently be viewed as an emerging hypothesis supported by biologic plausibility and partial mechanistic overlap with macrophage state transitions rather than a fully established causal pathway.

Lactate can also modulate macrophage behavior and reduce pro-inflammatory signaling through receptor-mediated pathways [68,69]; these effects are mechanistically distinct from lactylation and are separated explicitly (Section 5.2).

#### 4.2.4 Cancer progression and drug resistance

Histone lactylation has been shown to influence cancer progression in various types of cancer, including ocular melanoma, colorectal cancer, and bladder cancer. This modification is associated with several pathophysiological processes, including liver fibrosis and immune responses [70]. Specifically, histone modifications, such as H3K9 lactylation and H4K12 lactylation, have been correlated with drug resistance [71]. Non-histone lactylation, which modifies proteins such as Yes-associated protein 1 (YAP), TEA domain 1 (TEAD1), and Nijmegen breakage syndrome 1 (NBS1), enhances cellular proliferation, DNA repair, chemotherapy resistance, and tumorigenesis [72].

The investigation by Li et al. [73] outlined that tumor-derived lactate promotes resistance to bevacizumab treatment in colorectal cancer by facilitating autophagy enhancer protein RUBCNL expression through histone H3 lysine 18 lactylation (H3K18la). This suggests that lactylation plays a crucial role in the maintenance of chemotherapy resistance by modulating key pathways associated with tumor progression.

Additionally, researchers have explored the possibility of manipulating lactylation processes as a therapeutic strategy. Targeting lactylation-related pathways could provide novel strategies for overcoming drug resistance in cancer, indicating lactylation as a promising biomarker for treatment resilience [74].

Lactylation's influence on DNA repair processes emphasizes the importance of understanding this post-translational modification in therapeutic contexts. For instance, lactylation of NBS1 promotes effective homologous recombination DNA repair, significantly impacting tumor response to chemotherapy by enhancing the efficiency of the DNA repair machinery [36].

While lactylation appears to facilitate cancer progression, including enhanced proliferation and metastasis, some studies also highlight its potential anti-cancer effects. Lactylation has been implicated in regulating pathways such as the mitogen-activated protein kinase (MAPK) pathway and transforming growth factor beta (TGF-β), as well as influencing apoptosis [34]. This duality in lactylation's effects emphasizes the complexity of targeting it within cancer therapy, indicating the need for context-specific therapeutic strategies [75].

We interpret divergent cancer outcomes using the framework in Section 1.3: cell type (tumor cell vs immune cell), metabolic context (acute stress vs chronic Warburg bias), compartmentalization (intracellular lactylation vs extracellular lactate signaling), and time/tumor stage likely determine whether lactylation supports immune evasion and resistance versus differentiation, vulnerability, or growth restraint. This supports a translational strategy focused on context-specific targeting rather than global inhibition.

#### 4.2.5 Trauma and critical illness (Integration with sepsis biology)

Trauma shares with sepsis early hypermetabolism, lactate accumulation, endothelial activation, and immune dysregulation that can evolve into immune paralysis or persistent inflammation [2,3].

Trauma-specific lactylation datasets remain less developed than sepsis and cancer; thus, we treat trauma lactylation biology as an emerging area where mechanistic extrapolation should be explicitly labeled.

Nonetheless, given shared metabolic-immune phenotypes, lactylation is a plausible candidate mechanism for shaping macrophage transitions, endothelial programs, and late immune trajectories after trauma, meriting dedicated future study with time-resolved sampling and site-specific validation.

## 5 Methodological and translational considerations

### 5.1 Methodological considerations and technical limitations

Lactylation research faces recurring technical constraints that affect interpretation: (1) low modification stoichiometry relative to acetylation, making detection sensitive to enrichment thresholds [18,20]; (2) antibody specificity and potential cross-reactivity with other acyl marks; (3) mass spectrometry assignment challenges in complex samples and the need for robust fragmentation/site localization; (4) compartmentalization (low lactoyl-CoA abundance) complicating inference about dominant writer mechanisms [20]; and (5) causality gaps where correlations between lactate and lactylation are not matched by site-specific functional testing (e.g., mutagenesis or writer/eraser perturbation).

### 5.2 Translational perspective: Driver, biomarker, or both?

Current evidence supports lactylation as a plausible driver in select contexts with mechanistic depth (e.g., HMGB1 modification cascades in sepsis models) [23,51]; lactylation-linked DNA repair programs in cancer resistance [36]).

Lactylation is also proposed as a biomarker axis in some settings (e.g., histone H3K18 lactylation in septic shock severity proposals) [50], but clinical translation faces constraints: assay sensitivity and specificity in blood/tissue, sample handling, turnaround time in acute care, and discrimination of lactylation from related acyl marks.

Therapeutic targeting is challenging because broad inhibition of writers/erasers risks disrupting adaptive responses. A feasible strategy will likely require (i) patient stratification by metabolic phenotype, (ii) time-window targeting, and (iii) node-specific approaches rather than global suppression of lactate biology.

## 6 Conclusion

Lactylation represents a mechanistically compelling link between metabolic stress and epigenetic/signaling outputs across sepsis, trauma, inflammation, wound environments, and cancer. Evidence is strongest in defined mechanistic systems (e.g., macrophage HMGB1 modification cascades in sepsis models and lactylation-linked DNA repair programs in cancer drug resistance), while other areas particularly trauma-specific actylation biology and lactylation-specific roles in wound repair remain emerging.

A central message is context: lactylation may be adaptive during early stress responses yet pathological when sustained, mislocalized, or coupled to chronic glycolytic bias. The field now requires time-resolved, cell-type resolved, and site-specific testing to determine when lactylation is a driver, a downstream consequence, or a biomarker correlate.

Priorities include (i) disease-model validation of dominant writer/eraser pathways, (ii) stoichiometry-aware site-specific functional testing, (iii) rigorous separation of lactate receptor signaling from lactylation-dependent effects, and (iv) development of clinically feasible assays and stratification frameworks. With these advances, lactylation may mature from an exciting mechanistic bridge to a practical diagnostic and therapeutic axis in critical illness, chronic inflammation, and oncology.

## Declaration of generative AI and AI-assisted technologies in the manuscript preparation process

During the preparation of this work the author(s) used Paperpal in order to edit for grammer and language. After using this tool/service, the author(s) reviewed and edited the content as needed and take(s) full responsibility for the content of the published article.

## Funding

This research did not receive any specific grant from funding agencies in the public, commercial, or not-for-profit sectors.

## CRediT authorship contribution statement

**David Bar-Or:** Conceptualization, Writing – original draft. **Kaysie Banton:** Writing – review & editing. **David Acuna:** Writing – review & editing. **Jason Williams:** Writing – review & editing. **Carlos H. Palacio:** Writing – review & editing. **Christopher Zaw-mon:** Writing – review & editing. **Raymond Garrett:** Writing – review & editing. **Tyler Crawley:** Writing – review & editing. **Daniel Paredes:** Writing – review & editing.

## Declaration of competing interest

The authors declare that they have no known competing financial interests or personal relationships that could have appeared to influence the work reported in this paper.

## Data Availability

No data was used for the research described in the article.
