# Corrigendum to “Lactylation as a metabolic–epigenetic switch: Mechanisms and roles in cancer, sepsis, trauma, inflammation, and tissue repair” [Biochem. Biophys. Rep. 45 (2026) 102507]

**DOI:** 10.1016/j.bbrep.2026.102541

**Published:** 2026-03-10

**Authors:** David Bar-Or, Kaysie Banton, David Acuna, Jason Williams, Carlos H. Palacio, Christopher Zaw-mon, Raymond Garrett, Tyler Crawley, Daniel Paredes

**Affiliations:** a Trauma Services, Swedish Medical Center, Englewood, CO, USA; b Trauma Services, McAllen Hospital, McAllen, Texas, USA; c Trauma Services, Wesley Medical Center, Wichita, KS, USA; d Trauma Services, Lutheran Hospital, Wheatridge, CO, USA; e Trauma Research, Swedish Medical Center, Englewood, CO, USA; f Knoebel Institute for Healthy Aging, University of Denver, Denver, CO, USA

The authors regret <Unfortunately a Co-Author after it was published informed us that we have the wrong affiliation for him. In the above article head I have corrected the affiliation and highlighted it. Thank you for your consideration>.

The authors would like to apologise for any inconvenience caused.

